# A Lipidomic Approach to Studying the Downregulation of Free Fatty Acids by Cytosolic Phospholipase A_2_ Inhibitors

**DOI:** 10.3390/biom15050626

**Published:** 2025-04-27

**Authors:** Asimina Bourboula, Christiana Mantzourani, Ioanna Chalatsa, Christina Machalia, Evangelia Emmanouilidou, Maroula G. Kokotou, George Kokotos

**Affiliations:** 1 Department of Chemistry, National and Kapodistrian University of Athens, 15771 Athens, Greece; minabour@chem.uoa.gr (A.B.); chrmantz@chem.uoa.gr (C.M.); 2 Center of Excellence for Drug Design and Discovery, National and Kapodistrian University of Athens, 15771 Athens, Greece; 3 Laboratory of Biochemistry, Department of Chemistry, National and Kapodistrian University of Athens, 15771 Athens, Greece; ioannachalatsa@yahoo.gr (I.C.); cmachali@chem.uoa.gr (C.M.); eemman@chem.uoa.gr (E.E.); 4 Laboratory of Chemistry, Department of Food Science and Human Nutrition, Agricultural University of Athens, Iera Odos 75, 11855 Athens, Greece

**Keywords:** fatty acids, inhibitors, LC-HRMS, lipidomics, phospholipase A_2_

## Abstract

Inhibitors of cytosolic phospholipase A_2_ (GIVA cPLA_2_) have received great attention, since this enzyme is involved in a number of inflammatory diseases, including cancer and auto-immune and neurodegenerative diseases. Traditionally, the effects of GIVA cPLA_2_ inhibitors in cells have been studied by determining the inhibition of arachidonic acid release. However, although to a lesser extent, GIVA cPLA_2_ may also hydrolyze glycerophospholipids, releasing other free fatty acids (FFAs), such as linoleic acid or oleic acid. In the present work, we applied a liquid chromatography–high-resolution mass spectrometry method to study the levels of intracellular FFAs, after treating cells with selected GIVA cPLA_2_ inhibitors. Six inhibitors belonging to different chemical classes were studied, using SH-SY5Y neuroblastoma cells as a model. This lipidomic approach revealed that treatment with each inhibitor created a distinct intracellular FFA profile, suggesting not only inhibitory potency against GIVA cPLA_2_, but also other parameters affecting the outcome. Potent inhibitors were found to reduce not only arachidonic acid, but also other long-chain FAs, such as adrenic or linoleic acid, even medium-chain FAs, such as caproic or caprylic acid, suggesting that GIVA cPLA_2_ inhibitors may affect FA metabolic pathways in general. The downregulation of intracellular FFAs may have implications in reprogramming FA metabolism in neurodegenerative diseases and cancer.

## 1. Introduction

**Figure 1:**
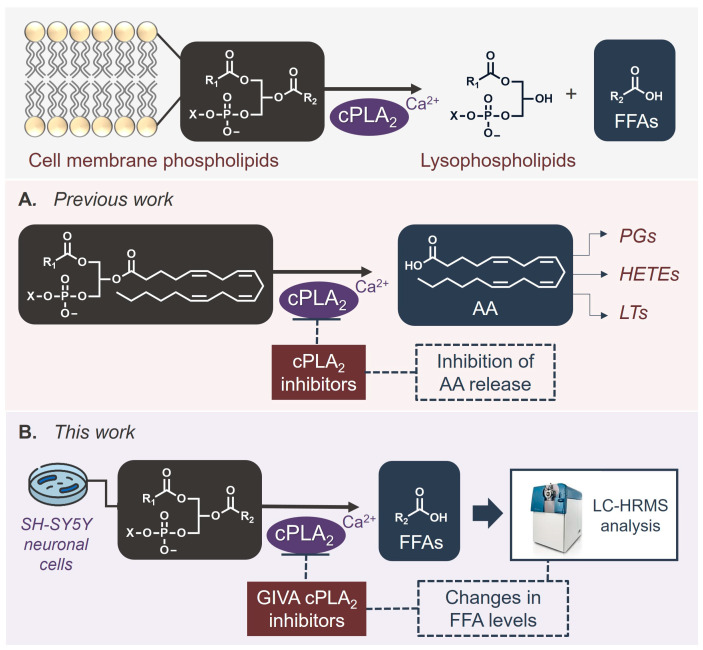
Enzymatic hydrolysis of glycerophospholipids by GIVA cPLA_2_. Generation of arachidonic acid and inhibition of its release by GIVA cPLA_2_ inhibitors (previous work, (**A**)). Study of intracellular FFA levels, following treatment of SH-SY5Y cells with GIVA cPLA_2_ inhibitors, by liquid chromatography–high-resolution mass spectrometry (this work, (**B**)).

Phospholipases A_2_ (PLA_2_s) is a superfamily of enzymes able to catalyze the hydrolysis of the ester bond at the *sn*-2 position of glycerophospholipids, resulting in the release of free fatty acids (FFAs) and lysophospholipids (Figure 1) [1,2,3]. Among the various groups and subgroups of PLA_2_s, cytosolic phospholipase A_2_ (GIVA cPLA_2_, also referred to as cPLA_2_α) is the most well-studied enzyme [4,5]. This particular lipolytic enzyme shows a pronounced preference for the hydrolysis of arachidonic acid from membrane phospholipids, whose release initiates the generation of numerous bioactive eicosanoids that are involved in various pathological conditions [6]. The involvement of GIVA cPLA_2_ in diverse inflammatory diseases has rendered it an attractive target for the development of novel anti-inflammatory medicines, and as a consequence, a great variety of synthetic small-molecule GIVA cPLA_2_ inhibitors have been developed over the years in both academia and pharma sectors [7,8,9].

Arachidonic acid (AA) is the precursor of various lipid signaling eicosanoids, such as prostaglandins and leukotrienes [6]. These eicosanoids are generated from arachidonic acid through three distinct metabolic pathways: the cyclooxygenase, lipoxygenase, and cytochrome P450 pathways, and they act, in general, as proinflammatory mediators [6,7,10,11]. Since the eicosanoids generated from the arachidonic acid metabolism are involved in diverse inflammatory diseases, the regulation of arachidonic acid production may contribute to the management of inflammatory-related diseases.

In addition to the attention paid to arachidonic acid and the resulting eicosanoids, due to their involvement in inflammation, fatty acids (FAs), in general, have received a lot of attention, due to their implications in cancer and neurological disorders. FAs are not only structural components of membranes, but they also play roles as secondary messengers and, certainly, as fuel sources for energy production. Thus, recent interest has been focused on clarifying the role of FAs in chemoresistance and the reprogramming of their metabolism in cancer [12,13].

Dysregulation of lipid metabolism has also been implicated in the pathogenesis of neurodegenerative disorders [14,15]. Alzheimer’s disease (AD), which is characterized by memory decline and cognitive dysfunction, is related to abnormalities in sphingolipid and glycerophospholipid metabolism, while in Parkinson’s disease (PD), which is characterized by motor dysfunction, muscle rigidity abnormalities, and slow gait, abnormal lipid metabolism leads to the accumulation of α-synuclein and the dysfunction of mitochondria and the endoplasmic reticulum. One of the hallmarks of PD and related synucleinopathies is the deposition of α-synuclein aggregates generated by misfolded conformers of the protein. Numerous lipid molecules, including FAs, may interact with α-synuclein [16,17]. In particular, polyunsaturated FAs (PUFAs), such as arachidonic acid or docosahexaenoic acid (DHA), have been reported to interact with α-synuclein, resulting in its oligomerization and subsequent cytotoxicity [18,19,20].

We have recently shown that treating neuroblastoma SH-SY5Y cells with the GIVA cPLA_2_ inhibitor GK200 reduced the level of arachidonic acid, resulting in a remarkable reduction in the levels of both oligomeric and monomeric α-synuclein, and thus promoting neuronal cell survival [21]. Most recently, we developed second-generation thiazolyl ketone inhibitors of GIVA cPLA_2_, and we demonstrated that inhibition of GIVA cPLA_2_ causes oxidative stress-dependent cell death in acute leukemia cells [22].

Given the specificity of GIVA cPLA_2_ for hydrolyzing arachidonic acid substrates, the effect and the potency of a particular GIVA cPLA_2_ inhibitor in cells is traditionally studied by monitoring the inhibition of arachidonic acid release (Figure 1A). However, for studies in cells and in vivo, it would be of great importance to monitor the effect caused by a GIVA cPLA_2_ inhibitor not only on a single lipid molecule, such as arachidonic acid, but on a number of potential bioactive lipid molecules. Such a functional lipidomics approach [23,24] may broaden our understanding of intracellular metabolic changes upon treatment of cells with GIVA PLA_2_ inhibitors.

The aim of this work was to develop a method able to provide information for the changes that happen in the levels of various FFAs in a cellular environment, using SH-SY5Y cells, a cancer cell line originating from human neuroblastoma, as a model (Figure 1B). Several potent GIVA cPLA_2_ inhibitors, including our recently developed thiazolyl ketone inhibitors [22], have been selected for our study. We present, herein, a liquid chromatography–high-resolution mass spectrometry (LC-HRMS) lipidomic approach, allowing for the study of a full set of FFAs in cells treated with GIVA cPLA_2_ inhibitors.

## 2. Materials and Methods

### 2.1. Chemicals and Reagents

All solvents used were of LC-MS analytical grade. Chloroform, methanol and dimethyl sulfoxide were purchased from Fisher Scientific (Laughborough, UK) and formic acid 98–100% from Chem-Lab (Zedelgem, Belgium). Tris-HCl, NaCl, SDS, NP-40 and EDTA were all molecular biology grade and purchased from Merck (Darmstadt, Germany). Caproic acid was purchased from Alfa Aesar (>98%, Lancashire, UK); caprylic acid, capric acid, myristic acid, myristoleic acid, pentadecanoic acid, margaric acid, linoleic acid, linolenic acid, arachidonic acid, behenic acid, *cis*-7,10,13,16-docosatetraenoic acid, and 4,7,10,13,16,19-*cis*-docosahexaenoic acid from Sigma Aldrich (>98%, Steinheim, Germany); lauric acid from Acros Organics (>99%, Geel, Belgium); palmitic acid, 9-palmitoleic acid, stearic acid, oleic acid, and 5,8,11,14,17-*cis*-eicosapentanoic acid from Fluka (analytical standard, Karlsruhe, Germany); and 10-*Z*-heptadecenoic acid, arachidic acid, bihomo-γ-linolenic acid, 7,10,13,16,19-*cis*-docosapentaenoic acid, and lignoceric acid from Cayman Chemical Company (>98%, Ann Arbor, MI, USA).

### 2.2. GIVA cPLA_2_ Inhibitors

The inhibitors GK420, GK427, GK470, and GK484 were synthesized in the Laboratory of Organic Chemistry, National and Kapodistrian University of Athens. Pyrrophenone and CAY10502 were purchased from Cayman Chemical Company (>98%, Ann Arbor, MI, USA). All inhibitors were diluted in dry dimethyl sulfoxide (DMSO) at a concentration of 5 mM, aliquoted and stored at −20 °C.

### 2.3. Cell Culture and Treatment with GIVA cPLA_2_ Inhibitors

Human neuroblastoma SH-SY5Y cells [25] were cultured in RPMI 1640 medium containing 10% (*v*/*v*) heat-inactivated fetal and bovine serum (FBS), 1% antibiotic/antimycotic (10,000 units/mL of penicillin, 10,000 μg/mL of streptomycin, and 25 μg/mL of amphotericin B), and 1% L-glutamine. Cells were maintained at 37 °C in a humidified 5% CO_2_ environment.

For the treatment with GIVA PLA_2_ inhibitors, cells were plated in 60 mm dishes and were treated with 3.5 μM of each inhibitor at 80% confluency for 24 h at 37 °C. Following treatment, cells were lysed in 100 μL lysis buffer containing 50 mM Tris-HCl (pH 7.6), 150 mM NaCl, 0.1% SDS, 1% NP-40, and 2 mM EDTA. After 20 min incubation on ice, cell lysates were prepared by centrifugation at 11,000× *g* for 5 min at 4 °C. The protein content in cell homogenates was estimated using the Bradford assay.

### 2.4. MTT Assay

SH-SY5Y cells were seeded in sterile tissue culture 96-well plates at a density of 10,000 cells per well. After 24 h, the medium was removed and replaced with fresh media containing 3.5 μΜ of each inhibitor, and the cells were further incubated for 24 h at 37 °C. DMSO was used as a vehicle at ≤0.03% (the maximum concentration, 3.5 μM, corresponds to 0.03% DMSO). The culture medium containing the compounds (or DMSO) was removed, and 100 μL of 0.5 mg/mL MTT reagent (Applichem, Darmstadt, Germany) diluted in RPMI medium was added to the cells for 3 h at 37 °C. After 3 h incubation, MTT was removed and 200 μL of DMSO was added. Absorbance values of formazan were measured at 540 nm using a BioTek Synergy H1 microplate reader (Agilent, Santa Clara, CA, USA). All compounds were tested in triplicate. For the nonlinear regression analysis, various concentrations of GK420 and GK484 (1.0, 3.5, 7.0, 15, 25, 45, 65, 85, 115, and 135 μΜ) were tested with the MTT assay as described above. The results were statistically analyzed using GraphPad PRISM Version 9 (GraphPad Software, Boston, MA, USA) to extract IC_50_ values.

### 2.5. Sample Preparation for Analysis

Chloroform (400 μL) and methanol (100 μL) were added to a sample of cell lysate (100 μL, prepared as described in Section 2.3) in a screw cap glass centrifuge tube. The sample was vortexed for about 30 s and then centrifuged at 4000× *g* for 10 min. The chloroform layer was collected, dried under argon (Ar), and re-dissolved in methanol/water 1:1 (100 μL) in a vial, and this mixture was used for the LC-MS/MS analysis.

### 2.6. Instrumentation

LC-MS/MS measurements were performed using an ABSciex Triple TOF 4600 (ABSciex, Darmstadt, Germany) combined with a micro-LC Eksigent, with an autosampler set at 5 °C and a thermostated column compartment (Eksigent, Darmstadt, Germany). Electrospray ionization (ESI) in negative mode was used for the MS experiments. The data acquisition method consisted of a TOF-MS full scan *m*/*z* 50–850 and several IDA-TOF-MS/MS (Information-Dependent Acquisition) product ion scans using 40 V Collision Energy (CE), with 15 V CES (Collision Energy Spread) used for each candidate ion in each data acquisition cycle (1091). This workflow allowed for quantitation (using TOF-MS primarily) and confirmation (using IDA-TOF-MS/MS) in a single run. Halo C18 2.7 μm, 90 Å, 0.5 × 50 mm^2^ from Eksigent (Darmstadt, Germany) was used as a column, and the mobile phase consisted of a gradient (A: H_2_O/0.01% formic acid; B: acetonitrile/0.01% formic acid/isopropanol 80/20 *v*/*v*). The elution gradient adopted started with 5% of phase B for 0.5 min, gradually increasing to 98% in the next 7.5 min. These conditions were kept constant for 0.5 min, and then the initial conditions (95% solvent A, 5% solvent B) were restored within 0.1 min to re-equilibrate the column for 1.5 min for the next injection (flow rate 55 µL/min). The data acquisition was carried out with MultiQuant 3.0.2 and PeakView 2.1 from AB SCIEX (Darmstadt, Germany).

EICs were obtained with the use of MultiQuant 3.0.2 (ABSciex, Darmstadt, Germany), which creates the base peak chromatograms for the masses that achieve a mass accuracy window of 5 ppm. The relative tolerance of the retention time window was set lower than ±0.2 min.

The list of fatty acids, together with their exact masses [M-H]^−^, their retention time R_t_ (min), and their limits of detection (LOD) and quantification (LOQ) [26], are shown in the Supplementary Materials (Table S1).

### 2.7. Method Validation

The guidelines of the EU Commission decision 2002/657/EC were followed to verify the accuracy and the precision of the method. SH-SY5Y cell lysate was spiked with a mixed standard solution of all analytes at three different concentrations (50 ng/mL, 200 ng/mL and 500 ng/mL, three replicates for each fortification level) to estimate the recovery (%R), the relative standard deviation (intra-day %RSD_r_ and inter-day %RSD_R_) and the matrix factor (MF). As shown in Table S2 (Supplementary Materials), satisfactory recoveries indicate the accuracy of the proposed method, while the precision was investigated by means of %RSD. The matrix factor was calculated as the ratio of the peak response in the presence of a matrix to the peak response in the pure solvent. Matrix factor values < 1 and >1 denote signal suppression and signal enhancement, respectively.

## 3. Results

### 3.1. Selection of GIVA cPLA_2_ Inhibitors

**Figure 2:**
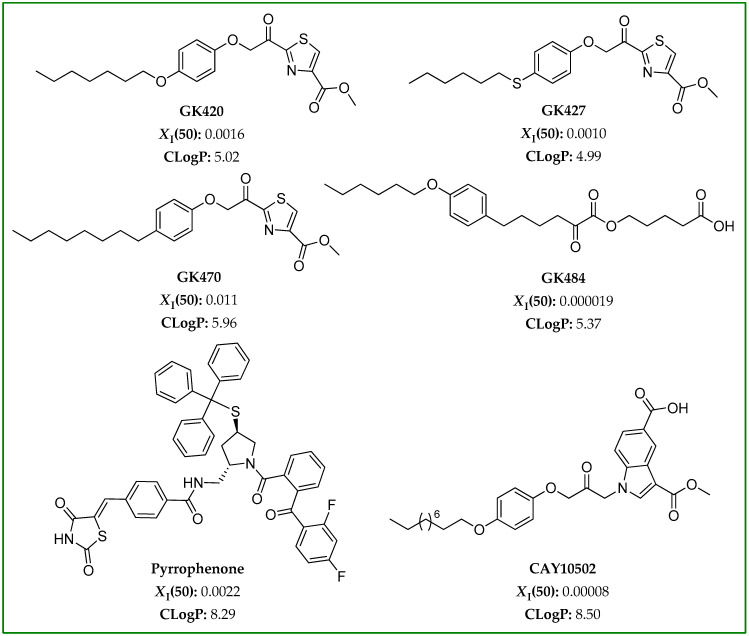
Structures of GIVA cPLA_2_ inhibitors used in this work, together with their *X*_I_(50) and ClogP values [22,27,28,29,30,31].

To study the effect of GIVA cPLA_2_ inhibitors on FFA levels in a cellular environment, we chose, in the present work, six inhibitors with diverse reactive functionalities. Among the various existing GIVA cPLA_2_ inhibitors, we first focused our attention on two of the thiazolyl ketones most recently developed by us, GK420 and GK427 (Figure 2) [22]. Both of them are quite potent inhibitors, exhibiting *X*_I_(50) values of 0.0016 and 0.0010 [22], respectively. *X*_I_(50) is defined as the mole fraction of the inhibitor in the total substrate interface required to inhibit the enzyme activity by 50%, and the *X*_I_(50) values were determined using a group-specific radioactivity-based mixed micelle assay. The above second-generation thiazolyl ketone inhibitors were designed starting from GK470 (also known as AVX235) (Figure 2), which has been reported to exhibit in vivo anti-inflammatory effects both in a preventative and a curative collagen-induced arthritis model [27]. GK470, presenting *X*_I_(50) 0.011 [27], was included in the study for comparison. A highly potent inhibitor belonging to the 2-oxoesters family, GK484 (Figure 2), was also selected for this study. This inhibitor, previously developed by us, exhibits *X*_I_(50) 0.000019 [28]. Furthermore, we selected two additional GIVA cPLA_2_ inhibitors, which belong to different chemical classes, possessing entirely different structural motifs. The pyrrolidine-based inhibitor pyrrophenone (Figure 2), developed by the pharmaceutical company Shionogi [29], presents *X*_I_(50) 0.008 [30], and it has been used in various in vitro and in vivo studies. The 1-heteroarylpropan-2-one inhibitor CAY10502 (Figure 2), developed by Lehr et al. [31], is a highly potent inhibitor of GIVA cPLA_2_ (*X*_I_(50) 0.00008). All the inhibitors used in this work are selective inhibitors of GIVA cPLA_2_.

GIVA cPLA_2_ acts at the lipid/water interface, and its synthetic inhibitors usually possess high lipophilicity, which means high positive ClogP values. The ClogP value provides a measure of the hydrophobicity of an inhibitor, and represents the calculated partition coefficient in octanol/water on a logarithmic scale. Taking into consideration Lipinski’s rule of five [32], small-molecule inhibitors with ClogP values higher than 5 are not expected to present favorable ADME (absorption, distribution, metabolism, and excretion) properties. The ClogP values were calculated for all the inhibitors used in the present study, using ChemOffice Ultra 11.00 (CambridgeSoft, Cambridge, MA, USA), and are shown in Figure 2. The ClogP values for GK420, GK427, GK470, GK484, pyrrophenone, and CAY10502 are 5.02, 4.99, 5.96, 5.37, 8.29, and 8.50, respectively.

### 3.2. Treatment of SH-SY5Y Cells with GIVA cPLA_2_ Inhibitors and Determination of FFAs by LC-HRMS

**Figure 3:**
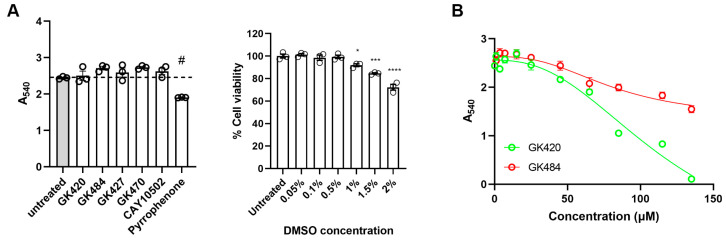
Effects of GIVA cPLA_2_ inhibitors on cell viability. (**A**) MTT assay for all compounds at 3.5 μΜ. Only pyrrophenone shows significant decrease in cell viability. Statistics obtained by Student’s *t*-test comparing cells treated with each inhibitor with cells treated with DMSO as vehicle (untreated). ^#^ *p* < 0.0001. DMSO alone had no effect on cell viability at concentrations used (<0.05%, left). Statistics by One-way Anova followed by Dunnett’s multiple comparison test (* *p* = 0.0224, *** *p* = 0.0001, **** *p* < 0.0001). (**B**) IC_50_ values obtained following treatment with GK420 or GK484 inhibitors, using nonlinear regression.

**Figure 4:**
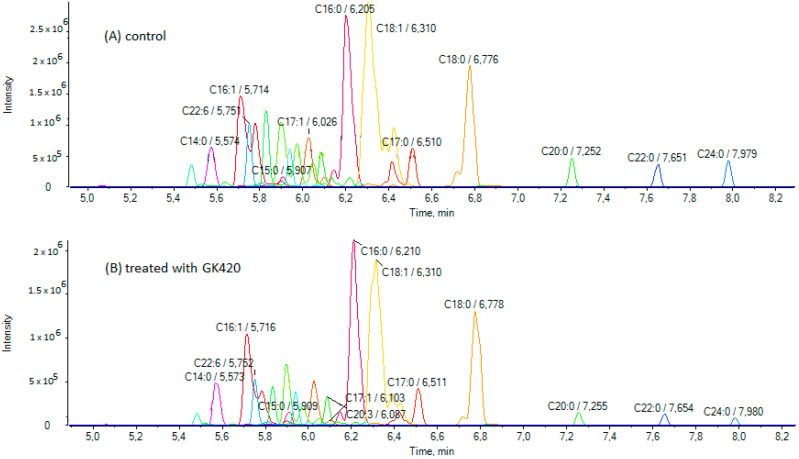
Extracted ion chromatograms (EICs) of FFAs in a representative control sample of SH-SY5Y cells (**A**) and a sample treated with the inhibitor GK420 (**B**).

SH-SY5Y cells were treated with each GIVA cPLA_2_ inhibitor at a concentration of 3.5 μM for 24 h, and compared with cells treated with DMSO as vehicle. DMSO alone had no effect on cell viability. None of the synthesized inhibitors were cytotoxic at this concentration, as confirmed by the MTT assay (Figure 3A). Only pyrrophenone showed a small reduction in cell viability (Figure 3A). It must be noted that pyrrophenone is usually used in cell experiments at concentrations up to 2 μM [33,34]. Furthermore, for two selected inhibitors, thiazolyl ketone GK420 and 2-oxoester GK484, the IC_50_ values were estimated and found to be 118.1 μM and 81.7 μM, respectively (Figure 3B). No other changes in cell morphology were observed after a 24 h incubation with the inhibitors. Following treatment, the cells were lysed, and the lysates were extracted by chloroform/methanol (4/1 *v*/*v*) and used for analysis. An LC-HRMS method previously developed by us for the determination of FFAs [26] was applied, and the contents of 24 FAs (medium-chain and long-chain, saturated and unsaturated) were measured both in control samples and samples treated with the inhibitors. Figure 4 illustrates extracted ion chromatograms (EICs) of FFAs in a representative control sample of SH-SY5Y cells (A) and a representative sample treated with inhibitor GK420 (B). EICs of FFAs in representative control samples of SH-SY5Y cells and samples treated with either inhibitor GK427 or inhibitor GK484 are depicted in Figures S1 and S2 (Supplementary Materials), respectively.

### 3.3. Effects of GIVA cPLA_2_ Inhibitors on Intracellular FFA Levels in SH-SY5Y Cells

The effects of the thiazolyl ketone inhibitors GK470, GK420, and GK427, as well as of the 2-oxoester inhibitor GK484, are summarized in Table 1.

As shown in Table 1, comparing the effect of the thiazolyl ketones GK470, GK420, and GK427, it is clear that GK420 and GK427 reduced the level of arachidonic acid (31.76% and 33.21%, respectively), while GK470 had a minimal effect (<10%). These reductions are in line with the in vitro inhibitory potency of these thiazolyl ketones against GIVA cPLA_2_ (*X*_I_(50) GK470 0.011, GK420 0.0016, and GK427 0.0010). Similar reductions were observed for adrenic acid by treatment with GK470, GK420, and GK427 (20.54%, 32.01%, and 48.52%, respectively). GK427 also significantly reduced the long-chain linoleic acid (34.79%) and bishomo-γ-linolenic acid (32.13%), while GK420 significantly reduced the long-chain myristoleic acid (30.28%) and arachidic acid (57.30%). Of interest is the finding that all the thiazolyl ketone inhibitors reduced the levels of medium-chain FAs C6:0, C8:0, and C10:0. In particular, GK427 reduced the levels of C8:0 and C10:0 by 51.06% and 38.29%, respectively. This is the first time that such a reduction effect of GIVA cPLA_2_ inhibitors on intracellular medium-chain FFAs has been described in the literature.

In the case of 2-oxoester inhibitor GK484 (Table 1), a remarkable reduction in both arachidonic acid and adrenic acid was observed (46.09% and 66.85%, respectively). These values are in agreement with the in vitro inhibitory potency of GK484 against GIVA cPLA_2_ (*X*_I_(50) 0.000019), which is more potent than the thiazolyl ketones GK420 and GK427. In addition, GK484 caused a remarkable reduction in the levels of a variety of long-chain FAs (lauric acid 47.09%, myristic acid 36.71%, margaric acid 37.15%, 10-Z-heptadecanoic 37.65%, stearic acid 33.28%, linoleic acid 32.99%, total linolenic acid 37.87%, arachidic 48.17%, bishomo-γ-linolenic acid 46.85%, EPA 34.08%, docosapentaenoic acid 30.52%, lignoceric acid 43.77%, and behenic acid 55.75%). As for the medium-chain FAs, only C6:0 was found to be decreased, by 48.19%.

The effects of pyrrophenone and CAY10502 are summarized in Table 2.

Both pyrrophenone and CAY10502 reduced the level of arachidonic acid in a similar manner (22.39% and 27.69%, respectively, Table 2). Such a reduction of arachidonic acid by CAY10502 at the cellular level does not seem to be in agreement with the high in vitro inhibitory potency of this inhibitor against GIVA cPLA_2_ (*X*_Ι_(50) 0.00008), suggesting that parameters other than the in vitro inhibitory potency significantly affect the intracellular effect. A small reduction in adrenic acid (14.72%) was observed with pyrrophenone, while no effect of CAY10502 on adrenic acid was recorded. The most notable reductive effect of pyrrophenone was found for medium-chain C10:0 (34.16%). In the case of pyrrophenone, an unexpected significant increase in the levels of a number of long-chain PUFAs was observed (EPA 61.42%, linolenic acid 30.12%, docosapentaenoic acid 28.69%, DHA 20.29%), suggesting that this inhibitor affects the metabolism of FFAs in general. CAY10502 was shown to significantly reduce the levels of long-chain oleic acid (43.49%), linolenic acid (36.16%), and bishomo-γ-linolenic (35.30%).

**Figure 5:**
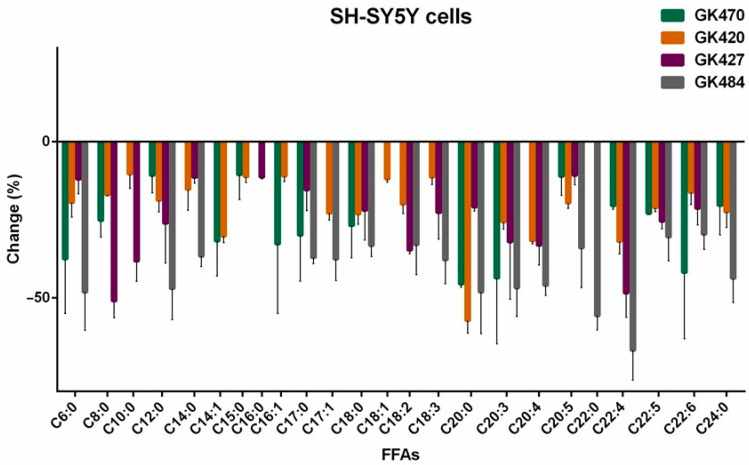
Change in intracellular FFAs upon treatment of SH-SY5Y cells with thiazolyl ketone (GK470, GK420, GK427) and 2-oxoester (GK484) inhibitors.

**Figure 6:**
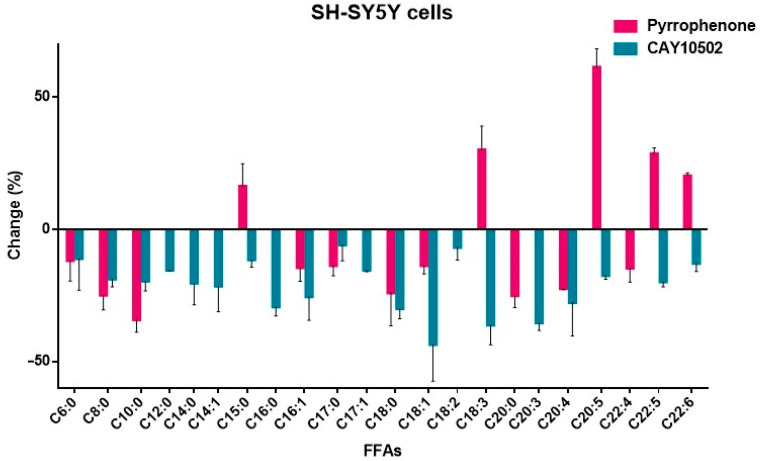
Change in intracellular FFAs upon treatment of SH-SY5Y cells with pyrrophenone and CAY10502.

The changes observed in the intracellular FFA levels, after treating cells with GIVA cPLA_2_ inhibitors, are better illustrated in Figure 5 and Figure 6.

## 4. Discussion

The development of small-molecule inhibitors of GIVA cPLA_2_ has been an active field of research for the last thirty years. Initially, the interest was focused on applying such inhibitors in treating inflammatory diseases, such as osteoarthritis and rheumatoid arthritis. Indole-based inhibitors, such as ecopladib and giripladib, entered clinical trials, but the trials were terminated when gastroenterological side effects were observed [35]. The topical application of GIVA cPLA_2_ inhibitors has also been explored. Another indole-based inhibitor, ZPL-5,212,372, has been studied in healthy adult volunteers and patients with moderate-to-severe atopic dermatitis, and found to improve symptoms [36]. The inhibitor AVX001, developed by Avexxin (now Coegin Pharma), has been studied for treating psoriasis and actinic keratosis [37]. Of special interest is the inhibitor ASB14780, developed by Asubio Pharma, which has potential application in the treatment of nonalcoholic fatty liver diseases, including fatty liver and hepatic fibrosis [38]. However, so far, no GIVA cPLA_2_ inhibitors have reached the market.

Although it is generally accepted that GIVA cPLA_2_ shows distinct selectivity for arachidonic acid substrates, recent lipidomic studies to understand *sn*-2 acyl chain specificity have shown that GIVA cPLA_2_ may also hydrolyze, although to a lesser extent, 1-palmitoyl-glycerophosphocholine substrates, containing, at the *sn*-2 position, linoleic acid, oleic acid, or palmitic acid [39,40]. In addition, using lipidomic procedures, it has been shown that GIVA cPLA_2_ releases not only arachidonic acid, but also adrenic acid in macrophages [41]. Adrenic acid is a 22-carbon unsaturated FA, considerably less explored than arachidonic acid. However, recently, it has attracted interest [42], and it has been included within the top 25 eicosanoids for the diagnosis of metabolic dysfunction-associated steatotic liver disease (MASLD) [43].

GIVA cPLA_2_ inhibitors are traditionally studied for their ability to inhibit the generation of arachidonic acid. However, based on recent studies on the selectivity of GIVA cPLA_2_, inhibition of GIVA cPLA_2_ could also affect additional FAs. Recently, we studied the effect of an oxoester GIVA cPLA_2_ inhibitor in SH-SY5Y neuronal cells [21], and we found that treatment with this inhibitor resulted in a remarkable reduction in arachidonic acid and adrenic acid. The interaction of FAs with α-synuclein is crucial, because it affects the aggregation of this protein, leading to the generation of α-synuclein fibrils, a hallmark of PD and related synucleinopathies. This prompted us to develop an approach that allowed for the study of a large set of intracellular FFAs, following treatment with GIVA cPLA_2_ inhibitors.

Potent and highly potent GIVA cPLA_2_ inhibitors (GK420 *X*_I_(50) 0.0016, GK427 *X*_I_(50) 0010, GK484 *X*_I_(50) 0.000019, pyrrophenone *X*_I_(50) 0.0022, and CAY10502 *X*_I_(50) 0.00008) reduced intracellular arachidonic acid levels from 22.39% to 46.09%. The thiazolyl ketone inhibitors GK420 and GK427 caused a 32.01% and 48.52% reduction in adrenic acid, respectively, while in the case of the 2-oxoester inhibitor GK484, remarkable adrenic acid downregulation (66.85%) was observed. Thiazolyl ketones also affected other long-chain FAs in different ways: GK427 reduced linoleic acid (34.79%) and bishomo-γ-linolenic acid (32.13%), while GK420 reduced myristoleic acid (30.28%) and arachidic acid (57.30%). The 2-oxoester inhibitor GK484 downregulated a more extensive set of long-chain FAs (lauric acid 47.09%, myristic acid 36.71%, margaric acid 37.15%, 10-Z-heptadecanoic acid 37.65%, stearic acid 33.28%, linoleic acid 32.99%, total linolenic acid 37.87%, arachidic acid 48.17%, bishomo-γ-linolenic acid 46.85%, EPA 34.08%, docosapentaenoic acid 30.52%, lignoceric acid 43.77%, and behenic acid 55.75%). On the other hand, the inhibitor CAY10502 significantly downregulated long-chain oleic acid (43.49%), linolenic acid (36.16%) and bishomo-γ-linolenic acid (35.30%). In the case of pyrrophenone, an unusual increase in the levels of linolenic acid (30.12%), EPA (61.42%), docosapentaenoic acid (28.69%), and DHA (20.29%) was observed, which may be attributed to off-target effects, since it is known that pyrrophenone inhibits the release of calcium from the endoplasmic reticulum [33].

Comparing the results observed for each of the inhibitors studied, it becomes evident that the effect of each inhibitor on the levels of intracellular FFAs is different. Apart from the in vitro potency of each inhibitor against GIVA cPLA_2_ and the mode of enzyme–inhibitor interaction at the molecular level, several other parameters, such as physicochemical properties, membrane penetration ability, and metabolic stability, may have affected the outcomes. At the molecular level, the interaction of each inhibitor with the active site of GIVA cPLA_2_ may include hydrogen bonding and electrostatic or hydrophobic contact. The inhibitors GK420, GK427, and GK470 may interact with the enzyme’s active site through their activated thiazolyl ketone carbonyl and additional functionalities [22]. Similarly, GK484 and CAY10502 may interact with the active site through activated oxoester and propanoyl carbonyl functionalities, respectively. On the contrary, pyrrophenone binds to GIVA cPLA_2_, creating numerous hydrophobic interactions distally from the enzyme’s active site, as has been shown previously [44]. From another point of view, the ability of each inhibitor to assess the cellular environment, penetrating the membrane, is of high importance. Of special interest is the lipophilicity of each inhibitor. Pyrrophenone and CAY10502 possess high lipophilicity (ClogP 8.29 and 8.50, respectively). On the contrary, GK420, GK427, and GK484 possess significantly lower lipophilicity (ClogP 5.02, 4.99 and 5.37, respectively). Thus, inhibitors combining high in vitro potency and ClogP values around 5 may considerably reduce arachidonic acid, adrenic acid, and other FAs.

When designing a GIVA cPLA_2_ inhibitor as a drug candidate, it is essential to first ensure high inhibitory potency for cPLA_2_ and selectivity for other PLA_2_ groups (for example, secreted PLA_2_ and calcium-independent PLA_2_). Then, absorption issues must be considered, exploring whether the inhibitor follows Lipinski’s rule of 5 [32], in particular regarding the ClogP value. The metabolic stability and potential off-target effects of these inhibitors are difficult to predict, and require further experimentation. According to these general considerations, among the inhibitors studied in the present work, the thiazolyl ketones GK420 and GK427, as well as 2-oxoester GK484, combine high inhibitory potency, high selectivity, and satisfactory ClogP values. As we have recently shown, GK420 and GK427 present satisfactory human plasma stability too [22]. On the contrary, 2-oxoester GK484 presents limited human plasma stability [28]. The cytotoxicity studies carried out in this work for thiazolyl ketone GK420 and 2-oxoester GK484 showed a similar profile for both compounds. Overall, thiazolyl ketone GK420 seems to be a promising inhibitor for further pharmacological evaluation.

The downregulation of FFA levels may be of potential therapeutic interest for cancer. Accumulating data indicate that FA metabolism is closely related to carcinogenesis and the progression of cancer, suggesting that the diverse lipid metabolic pathways may become new therapeutic targets [45,46]. A recent study by Koundouros et al. indicated a connection of oncogenic *PIK3CA* with enhanced arachidonic acid metabolism via mTORC2-PKCζ-cPLA_2_ signaling. Inhibition of cPLA_2_ by small-molecule ASB14780 downregulated the generation of arachidonic acid and, in combination with dietary fat restriction, resulted in growth inhibition of mutant PIK3CA-bearing breast tumors [47]. Our study, which revealed that small-molecule GIVA cPLA_2_ inhibitors are indeed able to downregulate the generation of FFAs in a cellular environment, suggests that the simultaneous use of chemotherapeutic agents together with GIVA cPLA_2_ inhibitors might result in better therapeutic outcomes.

## 5. Conclusions

In conclusion, we applied an LC-HRMS method to analyze a full set of FFAs in SH-SY5Y neuroblastoma cells, after treating the cells with GIVA cPLA_2_ inhibitors. Six inhibitors belonging to different chemical classes were used to monitor changes in 24 intracellular FFAs. Following this lipidomic approach, we were able to observe not only a reduction in arachidonic acid levels, but also reductions in the levels of other long-chain unsaturated FAs, such as adrenic acid and linoleic acid, that may serve as signaling lipids. However, it must be noted that each inhibitor caused a distinct change in the FFA profile, suggesting that, in addition to in vitro inhibitory potency of the inhibitor and the mode of interaction of the inhibitor with the enzyme’s active site, additional parameters, such as the lipophilicity of the inhibitor, may affect the outcome. In any case, the ability of GIVA cPLA_2_ inhibitors to regulate the levels of FFAs may contribute to understanding mechanisms of neurodegenerative diseases and cancer and uncovering new treatments for such diseases.

## Figures and Tables

**Table 1:** Change in intracellular FFAs following treatment of SH-SY5Y cells with thiazolyl ketone (GK470, GK420, GK427) and 2-oxoester (GK484) inhibitors (*n* = 3).

Fatty Acid	GK470		GK420		GK427		GK484	
	Change (%)	SD	Change (%)	SD	Change (%)	SD	Change (%)	SD
Caproic acid (C6:0)	−37.56	17.54	−19.52	4.71	−12.07	4.71	−48.19	12.22
Caprylic acid (C8:0)	−25.28	5.29	−17.08	0.31	−51.06	5.30	<10	-
Capric acid (C10:0)	<10	-	−10.48	4.46	−38.29	6.47	<10	-
Lauric acid (C12:0)	−10.82	5.66	−18.84	3.71	−26.19	12.74	−47.09	9.91
Myristic acid (C14:0)	<10	-	−15.30	6.76	−11.51	1.84	−36.71	3.32
Myristoleic acid (C14:1)	−31.81	11.23	−30.28	2.13	<10	-	<10	-
Pentadecanoic acid (C15:0)	−10.62	7.95	−11.30	1.84	<10	-	<10	-
Palmitic acid (C16:0)	<10	-	<10	-	−11.38	0.58	<10	-
9-Palmitoleic acid (C16:1)	−32.79	22.29	−11.08	1.77	<10	-	<10	-
Margaric acid (C17:0)	−29.97	14.75	<10	-	−15.50	6.66	−37.15	1.93
10-*Z*-Heptadecenoic acid (C17:1)	<10	-	−22.93	2.23	<10	-	−37.65	6.88
Stearic acid (C18:0)	−26.90	10.39	−23.20	3.32	−22.06	9.46	−33.28	3.54
Oleic acid (C18:1)	<10	-	−11.99	0.99	<10	-	<10	-
Linoleic acid (C18:2)	<10	-	−20.02	3.09	−34.79	1.08	−32.99	9.62
Total Linolenic acid (C18:3)	<10	-	−11.36	2.38	−22.79	8.43	−37.87	7.65
Arachidic acid (C20:0)	−45.61	1.07	−57.30	4.09	−20.97	1.28	−48.17	13.31
Bishomo-γ-linolenic acid (C20:3)	−43.70	21.05	−25.76	2.23	−32.13	18.37	−46.85	9.09
Arachidonic acid (C20:4)	<10	-	−31.76	0.98	−33.21	6.34	−46.09	3.19
EPA (C20:5)	−11.12	6.19	−19.67	1.76	−10.83	3.03	−34.08	12.73
Behenic acid (C22:0)	<10	-	<10	-	<10	-	−55.75	4.54
Adrenic acid (C22:4)	−20.54	1.21	−32.01	3.97	−48.52	7.81	−66.85	9.54
Docosapentaenoic acid (C22:5)	−23.03	0.13	−21.21	1.26	−25.52	2.40	−30.52	7.70
DHA (C22:6)	−41.95	21.14	−16.31	3.80	−21.39	5.26	−29.67	4.83
Lignoceric acid (C24:0)	−20.48	9.46	−22.50	4.97	<10	-	−43.77	7.76

**Table 2:** Change in intracellular FFAs following treatment of SH-SY5Y cells with pyrrophenone and CAY10502 (*n* = 3).

Fatty Acid	Pyrrophenone		CAY10502	
	Change (%)	SD	Change (%)	SD
Caproic acid (C6:0)	−11.84	7.62	−11.04	11.93
Caprylic acid (C8:0)	−24.86	5.59	−18.75	2.92
Capric acid (C10:0)	−34.16	4.72	−19.51	3.77
Lauric acid (C12:0)	<10	-	−15.41	0.18
Myristic acid (C14:0)	<10	-	−20.33	8.11
Myristoleic acid (C14:1)	<10	-	−21.47	9.54
Pentadecanoic acid (C15:0)	+16.34	8.47	−11.52	2.76
Palmitic acid (C16:0)	<10	-	−29.31	3.39
9-Palmitoleic acid (C16:1)	−14.47	5.11	−25.44	8.89
Margaric acid (C17:0)	−13.71	3.81	−5.83	5.97
10-*Z*-Heptadecenoic acid (C17:1)	<10	-	−15.41	0.59
Stearic acid (C18:0)	−24.01	12.39	−29.92	3.78
Oleic acid (C18:1)	−13.69	3.14	−43.49	13.93
Linoleic acid (C18:2)	<10	-	−6.86	4.69
Total Linolenic acid (C18:3)	+30.12	8.93	−36.16	7.48
Arachidic acid (C20:0)	−25.12	4.43	<10	-
Bishomo-γ-linolenic acid (C20:3)	<10	-	−35.30	2.91
Arachidonic acid (C20:4)	−22.39	0.38	−27.69	12.56
EPA (C20:5)	+61.42	6.92	−17.42	1.42
Behenic acid (C22:0)	<10	-	<10	-
Adrenic acid (C22:4)	−14.72	5.19	<10	-
Docosapentaenoic acid (C22:5)	+28.69	2.15	−19.76	1.95
DHA (C22:6)	+20.29	1.13	−12.80	3.15
Lignoceric acid (C24:0)	<10	-	<10	-

## Data Availability

All data supporting this study are included in the article and Supplementary Materials.

## Supplementary Materials

The following supporting information can be downloaded at https://www.mdpi.com/article/10.3390/biom15050626/s1: Table S1: List of fatty acids together with their exact masses [M-H]^−^, their retention time R_t_ (min), and their limits of detection (LOD) and quantification (LOQ). Table S2: Accuracy (recovery %), precision data (intra-day %RSD_r_, inter-day %RSD_R_), and matrix factor (MF) in spiked SH-SY5Y cell lysate. Figure S1: Extracted ion chromatograms (EICs) of FFAs in a representative control sample of SH-SY5Y cells (A) and a sample treated with the inhibitor GK427 (B). Figure S2: EICs of FFAs in a representative control sample of SH-SY5Y cells (A) and a sample treated with the inhibitor GK484 (B). References cited in Supplementary Materials [26,48].
