# Supplementary material for: A Lipidomic Approach to Studying the Downregulation of Free Fatty Acids by Cytosolic Phospholipase A2 Inhibitors
Source: Biomolecules. 2025 Apr 27;15(5):626. doi: 10.3390/biom15050626 (PMC12108850; doi:10.3390/biom15050626)
Supplement: Supplementary file 1 [file biomolecules-15-00626-s001.zip › biomolecules-3496484-supplementary.pdf]

Supplementary Material

A Lipidomic Approach for Studying the Downregulation of Free Fatty Acids by Cytosolic Phospholipase A_2 Inhibitors

Asimina Bourboula[1,2], Christiana Mantzourani[1,2], Ioanna Chalatsa[3], Christina Machalia[3], Evangelia Emmanouilidou[3], Maroula G. Kokotou[4,*] and George Kokotos[1,2,*]

***Correspondence:** mkokotou@aua.gr; gkokotos@chem.uoa.gr; Tel.: +30-210-7274462

Table of contents

1. Table S1. List of fatty acids together with their exact masses $[M-H]^-$, their retention time R_t (min), and their limits of detection (LOD) and quantification (LOQ).
2. Table S2. Accuracy (recovery %), precision data (intra-day $\%RSD_r$, inter-day $\%RSD_R$) and matrix factor (MF) in spiked SH-SY5Y cell lysate.
3. Figure S1. Extracted ion chromatograms (EICs) of FFAs in a representative control sample of SH-SY5Y cells (A) and a sample treated with inhibitor GK427 (B).
4. Figure S2. EICs of FFAs in a representative control sample of SH-SY5Y cells (A) and a sample treated with inhibitor GK484 (B).

Table S1. List of fatty acids together with their exact masses $[M\text{-}H]^-$, their retention time R_t (min), and their limits of detection (LOD) and quantification (LOQ).

Fatty acid	$[M\text{-}H]^-$	R_t (min)	LOD (ng/mL)	LOQ (ng/mL)
Caproic acid (C6:0) [1]	115.0765	2.1	0.5	1.5
Caprylic acid (C8:0) [1]	143.1078	3.2	0.5	1.1
Capric acid (C10:0) [1]	171.1391	4.1	0.5	1.7
Lauric acid (C12:0) [1]	199.1704	4.9	0.6	1.8
Myristic acid (C14:0) [1]	227.2017	5.6	0.6	1.8
Myristoleic acid (C14:1 n-5) [1]	225.1850	5.1	0.6	1.8
Pentadecanoic acid (C15:0) [1]	241.2173	6.0	0.8	2.4
Palmitic acid (C16:0) [1]	255.2330	6.3	0.9	2.3
cis-9-Palmitoleic acid (C16:1 n-7) [1]	253.2173	5.8	1.6	4.8
Margaric acid (C17:0) [1]	269.2486	6.6	0.8	2.4
cis-10-Heptadecenoic acid (C17:1 n-7) [1]	267.2330	6.2	0.8	2.4
Stearic acid (C18:0) [1]	283.2643	6.8	0.9	2.8
Oleic acid (C18:1 n-9) [1]	281.2486	6.4	0.7	2.3
Linoleic acid (C18:2 n-6) [1]	279.2330	6.0	0.6	1.8
total-Linolenic acid (C18:3)[1]	277.2173	5.6	0.6	1.8
Arachidic acid (C20:0) [1]	311.2956	7.0	0.8	2.4
Dihomo-γ-linolenic acid (C20:3 n-6) [1]	305.2486	6.1	0.6	1.8
Arachidonic acid (C20:4 n-6) [1]	303.2330	5.9	0.6	1.8
cis-5,8,11,14,17-Eicosapentaenoic acid (C20:5 n-3) [1]	301.2173	5.6	0.6	1.8
Behenic acid (C22:0)[2]	339.3269	7.7	0.8	2.4
Adrenic acid (C22:4)[2]	331.2643	6.3	0.8	2.4
cis-7,10,13,16,19-Docosapentaenoic acid (C22:5 n-3) [1]	329.2486	6.1	0.4	1.2
cis-4,7,10,13,16,19-Docosahexaenoic acid (C22:6 n-3) [1]	327.2330	5.9	0.4	1.2
Lignoceric acid (C24:0)[2]	367.3582	8.0	0.5	1.4

References

1. Kokotou, M. G., Mantzourani, C., Kokotos, G. (2020). Development of a liquid chromatography-high resolution mass spectrometry method for the determination of free fatty acids in milk. *Molecules, 25*, 1548.
2. Mantzourani, C., Batsika, C. S, Kokotou, M. G., Kokotos, G. (2022). Free fatty acid profiling of Greek yogurt by liquid chromatography-high resolution mass spectrometry (LC-HRMS) analysis. *Food Research International*, *160*, 111751.

Table S2. Accuracy (recovery %), precision data (intra-day %RSD$_r$, inter-day %RSD$_R$) and matrix factor (MF) in spiked SH-SY5Y cell lysate.

Analyte	Spike level 50 ng/mL				Spike level 200 ng/mL				Spike level 500 ng/mL			
	Recovery (%R)	RSD$_r$ (%)	RSD$_R$ (%)	MF	Recovery (%R)	RSD$_r$ (%)	RSD$_R$ (%)	MF	Recovery (%R)	RSD$_r$ (%)	RSD$_R$ (%)	MF
C6:0	85	11.3	12.8	0.8	90	11.5	10.2	0.8	93	0.6	1.1	1.2
C8:0	101	0.5	1.3	0.8	93	3.1	5.3	1.1	101	5.2	6.4	0.8
C10:0	104	8.7	1.6	0.8	101	10.6	9.2	1.1	88	10.1	9.7	0.8
C12:0	109	6.1	6.0	1.3	103	3.9	6.9	1.1	101	11.4	10.2	1.0
C14:0	90	8.3	10.5	0.8	104	4.5	3.2	1.2	105	0.2	0.8	1.1
C14:1	98	9.0	4.7	0.8	97	2.9	6.1	0.8	98	2.7	2.5	0.8
C15:0	101	10.3	7.6	1.1	98	10.5	8.1	0.8	98	1.3	1.5	1.1
C16:0	81	10.0	12.7	1.2	108	3.7	5.7	1.2	102	14.4	10.1	1.0
C16:1	97	9.5	10.0	0.8	109	0.5	1.5	1.2	102	6.6	5.4	1.2
C17:0	87	10.5	6.3	1.2	98	11.8	10.2	1.1	97	4.9	3.1	1.0
C17:1	97	1.3	1.9	1.2	102	5.2	4.2	0.8	100	3.1	5.1	1.2
C18:0	81	7.3	8.8	1.2	101	8.5	6.5	1.1	85	10.7	11.2	1.2
C18:1	84	0.9	2.2	0.8	100	5.4	3.4	1.0	85	10.2	8.5	1.2
C18:2	86	5.5	3.8	1.2	91	6.2	2.2	0.8	96	0.7	1.0	1.0
total-C18:3	86	10.5	6.3	0.8	103	11.9	8.9	0.8	103	7.9	7.4	0.8
C20:0	86	9.4	7.3	0.8	98	10.6	7.3	0.8	103	4.7	4.3	0.8
C20:3	86	6.9	5.9	1.1	95	6.3	5.3	0.8	96	7.2	6.5	1.1
C20:4	81	10.7	10.0	1.2	102	4.0	1.0	0.8	108	11.7	12.1	1.2
C20:5	94	2.0	5.2	1.1	97	9.0	3.0	1.1	98	3.9	2.4	1.1
C22:0	87	3.5	3.2	0.8	94	11.9	7.2	0.9	87	3.8	5.2	0.8
C22:4	85	3.4	4.2	0.8	86	10.8	8.1	0.8	85	9.4	7.7	1.2
C22:5	88	1.1	1.9	1.1	92	10.7	7.5	0.9	91	7.5	9.2	1.1
C22:6	91	3.3	6.5	0.8	101	0.7	1.5	1.1	103	1.1	0.6	1.2
C24:0	90	2.0	4.6	1.1	100	11.2	5.2	1.2	95	2.9	3.5	0.9

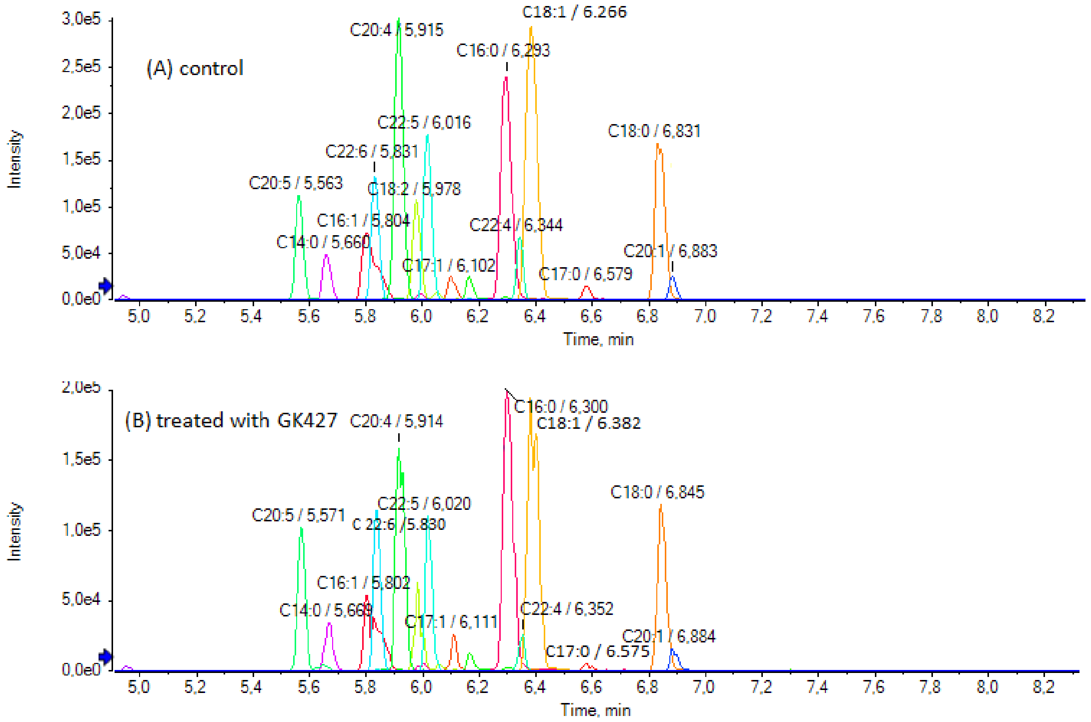

Figure S1. Extracted ion chromatograms (EICs) of FFAs in a representative control sample of SH-SY5Y cells (A) and a sample treated with inhibitor GK427 (B).

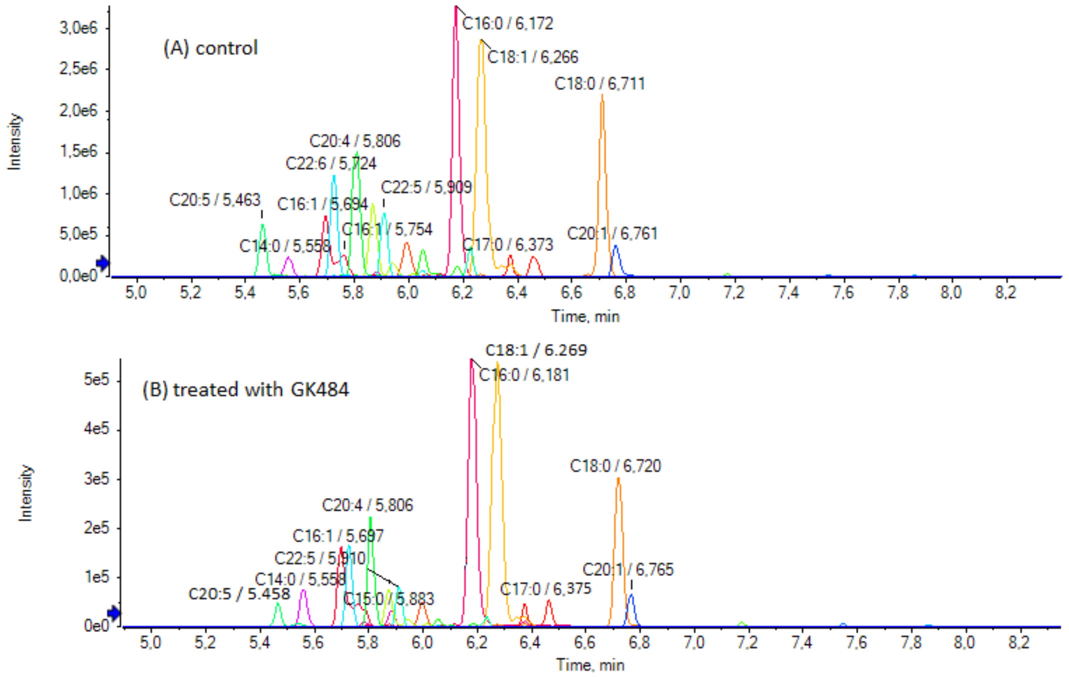

Figure S2. EICs of FFAs in a representative control sample of SH-SY5Y cells (A) and a sample treated with inhibitor GK484 (B).
